# Graphite shafts reduce forearm muscle activity in golf – a prospective case series of 40 right-handed amateur and professional golfers

**DOI:** 10.1186/s12891-026-09600-8

**Published:** 2026-02-11

**Authors:** Dominik Grieß, Kristian Nikolaus Schneider, Georg Gosheger, Christoph Theil, Jan Moritz Bochnia, Sebastian Bockholt

**Affiliations:** Department of Orthopaedics and Tumor Orthopaedics, University Hospital of Münster, Albert-Schweitzer Campus 1, 48149 Münster, Germany

**Keywords:** Epicondylitis, Shaft, EMG, Golf, Injuries, Overuse

## Abstract

**Background:**

Lateral and medial epicondylitis are common overuse injuries in golf affecting both amateur and professional players. Treatment options include medical but also non-medical interventions like optimizing golf equipment. While several studies have investigated the impact of grip size and style as well as the use of a golf glove on forearm muscle activity, the impact of shaft material remains unclear. Thus, aims of this study were (1) to assess the impact of different shaft material on the forearm muscle activity across the 5 phases of the golf swing, (2) to evaluate the effect on the individual performance and (3) to identify subgroups that may benefit more or less from this intervention.

**Methods:**

A cohort of 40 golfers (mean age: 51 years; mean handicap: 16), completed 5 golf swings with a steel and a graphite shaft, respectively. Muscle activity of the M. extensor carpi radialis brevis (ECRB), M. flexor carpi ulnaris (FCU), M. pronator teres (PT) and M. biceps brachii (BB) in both the lead and the trail arm were evaluated using surface electromyography (EMG). Golf performance was measured with a Trackman device (Trackmangolf, Vedbæk, Denmark). Subgroup analyses were performed with regard to gender, playing ability (handicap < 10 vs. ≥10), preexisting elbow pain during golf (visual analogue scale (VAS) < 2 vs. VAS ≥ 2) and grip style (overlap vs. interlock vs. baseball). Significance was set at *p* < 0.05.

**Results:**

During the final swing phase, the late follow-through, a graphite shaft leads to lower muscle activity in the FCU (*p* = 0.027) and the PT (*p* = 0.009) of the lead arm. Regarding grip style, the FCU of the trail arm showed higher muscle activity using a steel shaft (*p* = 0.021). In golfers without preexisting elbow pain, a steel shaft caused higher PT muscle activity in the lead (*p* = 0.002) as well as the trail arm (*p* = 0.032).

**Conclusion:**

Graphite shafts reduce forearm muscle activity in golf and may serve as a non-medical treatment for overuse injuries like lateral and medial epicondylitis.

**Trial registration:**

The study was registered in the German Clinical Trials Register as “The Impact of Shaft Materials on Upper Limb Muscle Activity in Golf” DRKS-ID: DRKS00036063. Retrospectively registered on February 3, 2025.

**Supplementary Information:**

The online version contains supplementary material available at 10.1186/s12891-026-09600-8.

## Introduction

Playing golf promotes a healthy lifestyle and golfers may increase their lifespan by up to five years compared to non-golfers [1, 2]. Regarding injury risk, golfers predominantly suffer from overuse pathologies like lateral and medial epicondylitis for which approximately 10% of all golfers require medical treatment [3–5]. Amateur players, in particular, have been found to be susceptible to elbow injuries and forearm muscle activities have been shown to vary amongst amateurs and professional players indicating differing injury patterns amongst both cohorts [3, 6, 7]. While substantial research has focused on various medical treatment options, relatively few studies have potential non-medical interventions: McHardy et al. have uncovered evidence that the club may contribute to the development of elbow injuries [4]. Sorbie et al. examined the impact of grip size but they did not find statistically significant differences in forearm muscle activity between different grip sizes [8]. Furthermore, Sorbie et al. assessed the impact of using a golf glove and found that wearing a glove while hitting a driver resulted in higher clubhead speed, ball speed and carry distance [9]. The impact of shaft material on forearm muscle activity has previously not been evaluated but may also contribute to overuse injuries like lateral and medial epicondylitis. Thus, the aims of this study are (1) to assess the impact of shaft material on forearm muscle activity across the five phases of the golf swing, (2) to evaluate its effect on performance and (3) to identify subgroups that may benefit more or less from either shaft material.

## Methods

Ethical approval was obtained from the local ethics committee (Ärztekammer Westfalen-Lippe; reference number: 2023-350-f-S). Written informed consent was obtained from all participants prior participation. The study was registered at the German Clinical Trials Register under the title “The impact of shaft materials on upper limb muscle activity in golf” with the registration number DRKS-ID: DRKS00036063 and retrospectively registered on February 3, 2025.

### Participants

Results of 40 golfers (9 female, 31 male) with a mean age of 51 years (range, 24–74 years), mean handicap of 16 (range, 0–30) and a mean golf experience of 15 years (range, 1–25 years) were available for analysis. All golfers were right-handed and used their right hand as the trailing hand.

Individual information on demographics, playing ability, grip style and preexisting elbow pain were obtained using a standardized questionnaire (Table 1).

**Table 1:** Demographic data

Variable		*n*	%
Sex			
Male		31	78
Female		9	23
Playing ability			
Amateur, HCP ≥ 10		9	23
Professional, HCP < 10		31	78
Grip Style			
Overlap		23	58
Interlock		14	35
Baseball		3	8
Elbow pain during golfing			
Golfers with elbow pain, VAS ≥ 2		12	30
Golfers without elbow pain, VAS < 2		28	70

*HCP* Handicap, *VAS* Visual analogue scale

### Apparatus

**Fig. 1:**
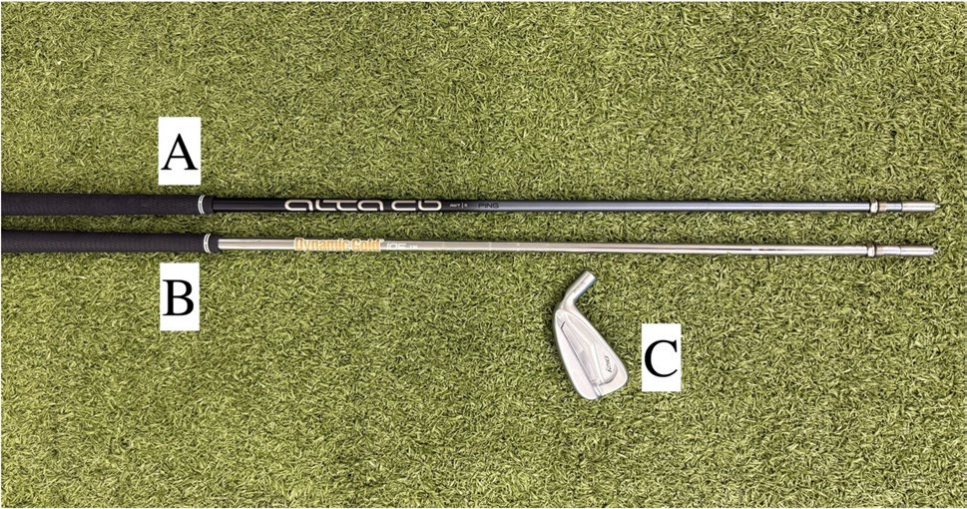
**A-C**: **A**: Graphite Shaft: PING Alta CB; **B**: Steel shaft: True Temper Dynamic Golf 105; **C**: Clubhead PING i210

The experimental setup was conducted inside a professional teaching studio at the Golf Club Royal St. Barbara’s in Dortmund, Germany. Golf swings were performed on an artificial turf golf mat without a tee. The hitting area was located 3,5 m from the net. The club used was a PING i210 (Ping Inc., Phoenix, USA). The fitting of the 7-iron is compatible with different shafts, using a specially modified screw system (Fig. 1).

For this study, shafts with a regular flex were used, all shafts had the exact length of 37 inch and were gripped with a standard GolfPride Tour Velvet golf grip (GolfPride, Pinehurst, USA). The graphite shaft PING Alta CB (Ping Golf, Phoenix, USA) had a weight of 72 g and torque of 2.1 (Fig. 1). The steel shaft True Temper Dynamic gold 105 (True Temper Sports, Memphis, USA) had a weight of 101 g and torque of 1.8 (Fig. 1).

The golf ball used for all golf swings was a Titleist Pro-V1 2023 (Acushnet Company, Fairhaven, USA). Performance was measured with the Trackman IV System (Trackmangolf, Vedbæk, Denmark) using the OERT-Technology (Optically Enhanced Radar Tracking). A 240fps slow-motion camera (iPhone 11, Apple, Cupertino, USA) was installed and synchronized with the EMG signal.

### Electromyography

**Fig. 2:**
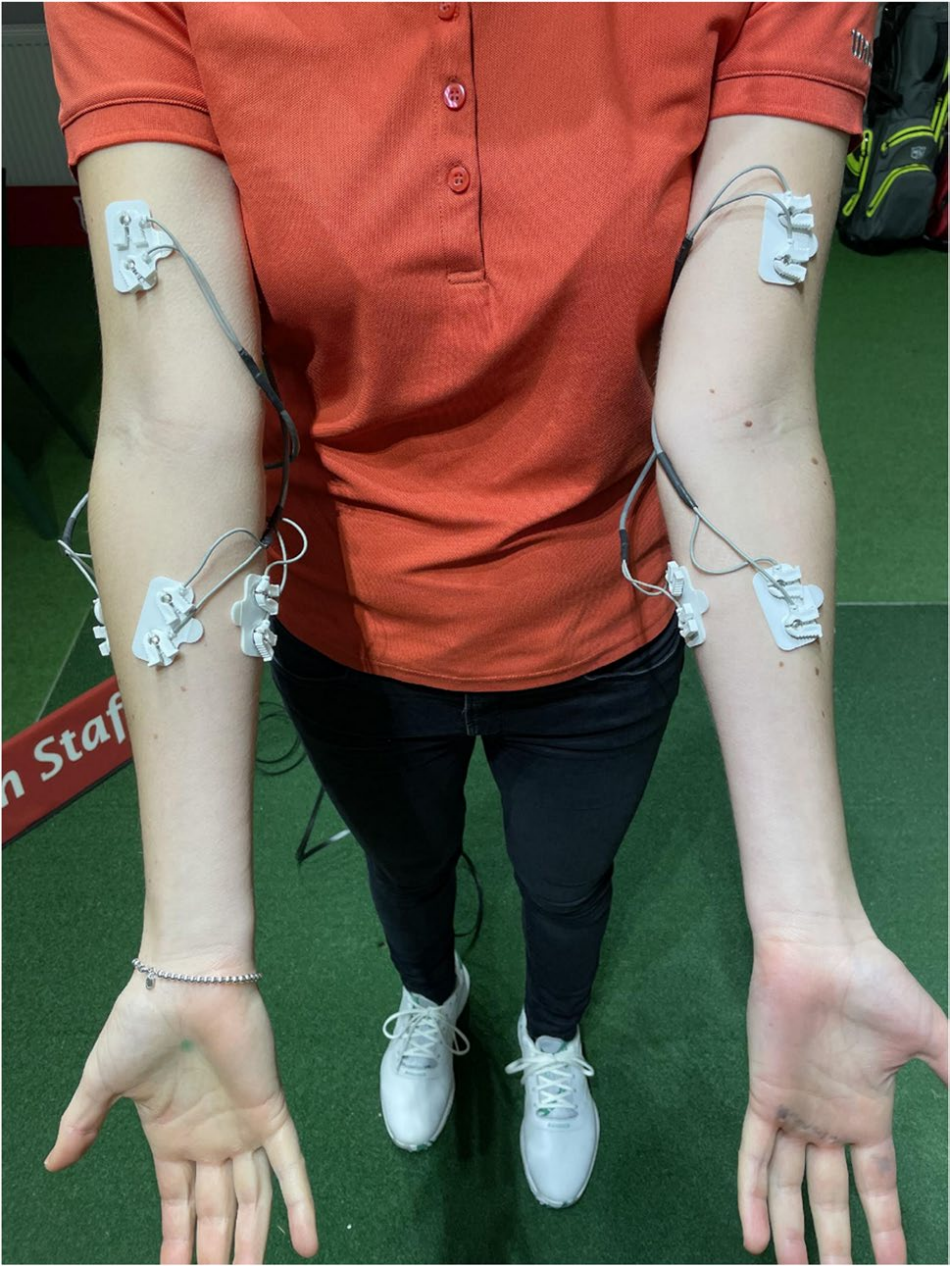
Surface EMG placed according to Bochnia et al. (2024) [10]

Surface EMG has previously been used to assess forearm muscle activity in golf [6–11]. The EMG system collected a total of 8 signals from surface EMG electrodes, measuring 4 muscles in each arm. The electrodes were placed on the M. flexor carpi ulnaris (FCU), M. extensor carpi radialis (ECRB), M. pronator teres (PT) and M. biceps brachii (BB) on the lead and trail arm, respectively (Fig. 2). The selection of muscles was based on their function and their previous use in the literature. The FCU represents the flexion and its tendon is crucially affected in medial epicondylitis. To expand on existing findings in the literature, the ERBC was selected to assess its role in wrist extension and radial deviation [10]. The PT with its origin at the epicondylus medialis demonstrates pronation. The BB muscle was selected to evaluate forearm flexion and supination.

In addition, a neutral electrode was placed on the Epicondylus lateralis of the trail arm. The study was set-up according to Bochnia et al. [10]: EMG signals were bandpass filtered at 50 Hz. The signals were transferred to the preamplifier which transmitted at 240 to 1400 Hz to the EMG base station. The rectified and smoothed EMG signal was digitally processed and subsequently employed for swing phase detection. The average and maximum muscle activity in µV was measured during the five phases of the golf swing. Absolute values were used in the analysis; no normalization was performed. All leads run through the golfer’s shirt and were connected with the preamplifier. The surface EMG electrode sites were cleaned and any obstructing hairs were shaved. In addition, the leads were securely fixed at the skin to stay in position.

### Golf swings

Participants were asked to run through their individual warm-up routine. Following the warm-up, participants were connected to the EMG system and performed 10 golf swings: 5 full golf swings were performed with the graphite and steel shafts, respectively. The swing sequence of both shafts was individually randomized (www.random.org, Dublin, Ireland). To avoid a drop in performance due to fatigue, a 45 s break was set between each golf swing.

### Statistical analysis

An a priori power analysis showed that with a total cohort of 40 golfers and an alpha of 0.05 a statistical power of > 0.99 can be reached. The obtained EMG data was recorded in an Excel sheet version 16.90.2 (Microsoft Corp., Redmond, WA, USA). For statistical analysis, the golf swing can be divided into five phases according to Jobe et al. [12]. Statistical analysis were conducted using SPSS Statistics version 29.0.2.0 (IBM Corp. Published 2023. IBM SPSS Statistics for MacOS, Version 29.0.2.0 Armonk, NY: IBM Corp). The Kolmogorov-Smirnov test was used to assess data distribution. The paired Wilcoxon signed rank test was performed. Additionally, subgroup analyses were conducted based on gender (male vs. female), grip style (overlap vs. interlocking vs. baseball), preexisting elbow pain (VAS < 2 vs. VAS ≥ 2) and playing ability (handicap < 10 vs. ≥10). Significance was set at *p* < 0.05.

## Results

### Shaft material

Comparing a steel to a graphite shaft, statistically significant deviations in forearm muscle activity were found for individual muscles during individual phases of the golf swing: The mean muscle activity of the PT and FCU of the lead arm were statistically significantly lower with a graphite shaft compared to a steel shaft during the late follow-through. Mean and maximum values of forearm muscle activities are given in Table 2.

**Table 2:** Mean and maximum muscle activity in µV during the five phases of the golf swing

Muscle	Takeaway		Forward Swing		Acceleration		Early follow-through		Late follow-through	
	Mean	Max.	Mean	Max.	Mean	Max.	Mean	Max.	Mean	Max.
Lead Arm										
ECRB										
Steel	88.93	150.71	62.47	83.61	84.30	105.72	145.78	185.38	139.20	248.53
Graphite	87.83	147.45	62.82	86.40	84.47	107.32	151.51	191.10	139.47	255.82
p-value	0.536	0.313	0.904	0.428	0.968	0.591	0.070	0.162	0.819	0.143
FCU										
Steel	93.92	163.60	152.26	184.66	155.21	174.62	185.05	222.85	158.17	268.86
Graphite	91.91	16,059	148.74	181.97	152.72	174.79	182.35	213.03	150.67	264.59
p-value	0.259	0.237	0.452	0.657	0.405	0.962	0.428	0.211	0.027*	0.333
PT										
Steel	65.37	120.39	69.53	92.10	79.17	94.27	148.64	20,349	166.09	298.52
Graphite	63.05	113.97	65.46	89.65	81.81	97.14	145.67	195.57	155.00	291.22
p-value	0.104	0.116	0.545	0.717	0.648	0.270	0.528	0.168	0.009*	0.093
BB										
Steel	34.78	86.10	42.28	72.05	66.48	94.97	156.29	206.58	176.12	306.59
Graphite	37.13	92.88	45.47	75.73	67.20	92.94	154.05	208.14	175.54	311.30
p-value	0.113	0.186	0.221	0.432	0.591	1.000	0.747	0.536	0.563	0.638
Trail Arm										
ECRB										
Steel	93.91	179.98	91.92	127.28	66.48	78.00	103.06	137.14	121.44	217.36
Graphite	94.36	179.47	93.47	130.50	66.42	78.69	100.22	136.74	116.50	217.59
p-value	0.925	0.550	0.098	0.088	0.757	0.452	0.979	0.757	0.347	0.619
FCU										
Steel	60.36	96.83	68.49	102.24	131.45	164.98	217.29	259.09	162.68	299.01
Graphite	61.85	99.93	67.95	101.49	128.06	161.75	219.35	262.38	163.29	303.15
p-value	0.615	0.221	0.405	0.464	0.090	0.216	0.819	0.893	0.788	0.757
PT										
Steel	40.03	97.42	39.31	61.52	69.92	94.27	144.63	190.59	149.30	280.72
Graphite	40.73	99.70	39.90	62.85	70.95	97.14	148.79	192.87	144.80	276.01
p-value	0.460	0.383	0.861	0.497	0.727	0.270	0.253	0.586	0.179	0.460
BB										
Steel	43.95	105.84	57.36	81.10	66.48	86.65	140.11	191.35	160.27	303.85
Graphite	43.37	102.56	56.00	79.06	67.20	87.83	142.31	192.00	157.49	303.88
p -value	0.610	0.291	0.076	0.221	0.591	0.747	0.798	0.809	0.717	0.914

Statistically significant p-values are shown in bold, * indicates a statistically significant decrease in muscle activity with the graphite shaft

*ECRB* Musculus extensor carpi radialis brevis, *FCU* Musculus flexor carpi ulnaris,* PT* Musculus pronator teres, *BB* Musculus biceps brachii

### Performance

Golf performance showed to be different in professionals who had an overall higher clubhead speed using a graphite shaft compared to a steel shaft (p=0.011). For the total cohort, the Trackman data did not show any statistically significant difference in clubhead speed comparing steel and graphite shaft. In addition, carry distances were similar amongst the graphite shaft and the steel shaft for all subgroups (Table 3).

**Table 3:** Performance, trackman data

Variable		Clubheadspeed (mph)	Carry Distance (m)
All	Graphite	72	106
	Steel	72	107
	p-value	0.113	0.657
Sex			
Male	Graphite	76	115
	Steel	76	115
	p-value	0.150	0.739
Female	Graphite	61	77
	Steel	60	78
	p-value	0.594	0.953
Playing Ability			
Amateur, HCP ≥ 10	Graphite	69	97
	Steel	69	98
	p-value	0.710	0.445
Professional, HCP < 10	Graphite	85	139
	Steel	84	138
	p-value	0.011*	0.678
Grip Style			
Overlap	Graphite	71	106
	Steel	71	108
	p-value	0.503	0.584
Interlock	Graphite	77	115
	Steel	77	115
	p-value	0.109	0.875
Baseball	Graphite	59	65
	Steel	59	67
	p-value	0.593	1.000
Elbow pain during golfing			
Golfers with elbow pain, VAS ≥ 2	Graphite	70,67	101,03
	Steel	70,86	103,20
	p-value	0.875	0.480
Golfers without elbow pain, VAS < 2	Graphite	73,14	108,51
	Steel	72,72	108,81
	p-value	0.074	0.964

Statistically significant p-values are shown in bold, * indicates a statistically significant increase with the graphite shaft

*HCP* Handicap, *VAS* Visual analogue scale

### Subgroup analyses

Regarding handicap, amateur players showed lower mean (p=0.005) as well as maximum (p=0.012) muscle activity in PT of the lead arm during the late follow-through using the graphite shaft. The maximum value for amateurs in the ECRB of the trail arm in the forward swing was higher with the graphite shaft (p=0.027). In professionals, the mean muscle activity of the ECRB increased during the forward swing (p=0.038) of the trail arm while using the graphite shaft.

Regarding gender, female golfers showed lower maximum muscle activity in PT (p=0.028) during takeaway of the lead arm using the graphite shaft. In the trail arm of female golfers, lower mean values were observed in the ECRB (p=0.028) during the forward swing using the steel shaft. However, the maximum muscle activity of the ECRB (p=0.008) increased in females trail arm during the acceleration phase. Among male golfers, higher mean muscle activity in ECRB (p=0.015) was observed in the lead arm during the early follow-through. When using the graphite shaft, male golfers demonstrated lower mean muscle activity in BB (p=0.036) during the forward swing of the trial arm. In male golfers, the mean muscle activity in PT (p=0.014) and FCU (p=0.034) decreased during the late follow-through of the lead arm.

Regarding grip style, using the overlap grip resulted in lower mean muscle activity in FCU (p=0.021) and higher maximum value in ECRB (p=0.023) during the forward swing of the trail arm using the graphite shaft. Golfers using the interlock grip showed decreased mean muscle activity in the FCU (p=0.016), in the PT (p=0.022) in the late follow-through and decreased maximum muscle activity in the ECRB (p=0.035) in the takeaway of the lead arm. The graphite shaft produced higher mean values in ECRB (p=0.016) and BB (p=0.035) during the early follow-through of the lead arm when using the interlock grip.

Regarding preexisting elbow pain, golfers who reported a pain level above two on the VAS showed lower maximum muscle activity in the FCU (p=0.034) during the acceleration phase of the trail arm and lower maximum muscle activity in the PT (p=0.050) during the late follow-through of the lead arm while using the graphite shaft. Golfers without preexisting elbow pain produced lower mean muscle activity in PT of both the lead (p=0.002) and trail arm (p=0.032) in the late follow-through as well as in BB (p=0.011) of the trail arm during the forward swing using the graphite shaft. An increased mean muscle activity was observed in BB (p=0.032) in takeaway of the lead arm in golfers without preexisting elbow pain.

## Discussion

Main findings of this study were (1) the graphite shaft leads to lower mean muscle activity of the PT and FCU of the lead arm during the late follow-through phase. (2) Professionals had higher clubhead speed using the graphite shaft. (3) The subgroup analysis indicated that male golfers and those without preexisting elbow pain may benefit using a graphite shaft.

Compared to a steel shaft, the graphite shaft leads to lower mean muscle activity of the PT and FCU in the lead arm during the late follow-through phase. Prior studies disclosed elbow injuries are most effected by overuse [3, 4, 13]. In this regard, it should be noted that anomalous muscle activity is associated with an increased risk of overuse injuries [10, 14, 15]. Even though medial epicondylitis in golfers is mostly caused by overuse of the trail arm, using a graphite shaft can be a useful protective treatment to prevent injury in the lead arm [16–18]. High forces are generated during impact in particular, however reducing muscle activity in the late follow-through phase can also contribute to reducing accumulated muscle load [13]. Furthermore, shaft material has shown to be a potential risk factor for developing elbow pain in sports [19, 20]. In golf, a graphite shaft could be a viable non-medical intervention, however, the overall shaft weight must also be taken into consideration. Since a steel shaft is heavier, the additional effort required to slow down this mass during the late follow-through phase may contribute to an increased muscle activity observed during this phase of the golf swing.

Yang et al. observed higher swing speeds and longer shot distance using lighter shafts [21]. In our study, the professionals reached a higher clubhead speed using the graphite shaft. However, this effect was not observed across all participants. It is possible that this advantage applies primarily to skilled golfers with refined techniques to convert this hypothetical advantage into a real advantage of a higher clubhead speed. There is no advantage in the carry distance suggesting the shots may have been of lower quality. This implies that a higher clubhead speed does not necessarily result in an optimal shot and a lack of weight may interfere with the stability of the swing technique. It is likely that performance is influenced not only by shaft weight but also by other material properties.

The subgroup analysis revealed that male golfers and those with preexisting elbow pain showed lower muscle activity than their counterparts. By using a graphite shaft in male golfers, over 80% of American golfers would benefit from a reduction of the muscle activity [22]. The graphite shaft can also be used as a supplementary non-medical intervention for preexisting elbow problems. Interestingly, muscle activity in the trail arm increased in female golfers during the acceleration phase when using the graphite shaft. This could be due to the lower weight of the graphite shaft, requiring female players to prevent the club from scooping and to keep the club behind their hands. In contrast, the higher inertia of the steel shaft likely initiates a greater lag. It should be mentioned that the observed differences between men and women in muscle activity may well be related to anatomy. The evidence is controversial, thus the carrying angle (cubitus valgus) has been examined in relation to lateral epicondylitis [23, 24]. The decrease in PT of the lead arm during the takeaway is explained by weight being a crucial factor in female golfers. In the takeaway, the golfer needs to lift and rotate the club while the lead arm moved into pronation, which is controlled by the PT. Noticeably, the analysis of grip style revealed lower muscle activity while using the overlap grip with the graphite shaft in the FCU during forward swing in the trail arm. In the present study most golfers used the overlap grip. This suggests that most golfers may be at risk of developing epicondylitis medialis in the trail arm when using a steel shaft. On the other hand, ECRB muscle activity in the trail arm increased during the forward swing with the graphite shaft. However, the interlock grip indicated other results. The ECRB and BB of the lead arm showed greater muscle activity with the graphite shaft, while PT and FCU in the lead arm showed lower muscle activity. This suggests that the positioning of the hands, combined with the shaft material, may be a protective modification adaptation for many golfers.

Based on our findings, we recommend a protective usage of graphite to prevent epicondylitis medialis. Even though, as already mentioned, the incidence of medial epicondylitis is more common in the trail arm than in the lead arm. The PT, which has its origin in epicondylus medialis, caused lower muscle activity with the graphite shaft in both the lead and trail arm in golfers without preexisting elbow pain.

### Limitations

Several limitations need to be acknowledged. Firstly, findings should not be generalized as the shafts used in this study differed not only in material but also in weight and the impact on muscle activity cannot be fully attributed to the material alone. However, the shafts selected for this study are shafts that are widely used in the golf market and that come with the weight difference.

Secondly, the torque, the resistance to axial twisting, differs between steel and graphite shafts. The torque of the steel shaft is 1.8 degree and that of the graphite shaft is 2.1 degree. Previous studies revealed, that the higher the torque value, the more axial twisting of the shaft occurs [25]. Therefore, the difference in torque has a potential impact on the forearm muscle activity that needs to be considered when interpreting results and findings.

Thirdly, while surface EMG is the best choice for our study, it has its limitations. Measurement accuracy can be influenced by sweat and hair. Prior to positioning the surface electrodes, the skin was cleaned and hair removed. However, fine wire electrodes provide higher accuracy when measuring muscle activity in a single muscle [26]. While electrode placement was standardized, deviations due to individual anatomy cannot be excluded. The use of fine wire electrodes during high-speed movement has not been considered due to the risk of injuries and hematomas [27].

Fourthly, the subjects hit golf balls from the ground, simulating conditions on a golf course. The artificial turf golf mat generates different dimensions as opposed to real turf. Hitting a divot could modify the impact of ground hitting. Likewise, it has not been studied how hitting the ground before and after the ball changes the muscle activity in the forearm.

Fifthly, we did not subdivide the grip type (weak, normal, strong) in the analysis. The grip and wrist position influence wrist angles and wrist movement [28, 29]. Thus it could be concluded that the forearm muscles are also affected.

## Conclusion

The graphite shaft reduces the muscle activity in the forearm muscles PT and FCU of the lead arm in the late follow-through. In golfers without preexisting elbow pain, a steel shaft caused higher PT muscle activity in the lead as well as the trail arm in the late follow-through. Golfers with preexisting elbow pain showed lower muscle activity in the FCU of the trail arm and in the PT of the lead arm using the graphite shaft.

## Supplementary Information

Supplementary Material 1.

## Data Availability

The data that support the findings of this study are available from the corresponding author upon reasonable request.

## Abbreviations

ECRB: Musculus extensor carpi radialis brevis
FCU: Musculus flexor carpi ulnaris
PT: Musculus pronator teres
BB: Musculus biceps brachii
EMG: Electromyopgraphy
HCP: Handicap based on World Handicap System
VAS: Visual analogue scala
