# Supplementary material for: Graphite shafts reduce forearm muscle activity in golf – a prospective case series of 40 right-handed amateur and professional golfers
Source: BMC Musculoskelet Disord. 2026 Feb 11;27:175. doi: 10.1186/s12891-026-09600-8 (PMC12930853; doi:10.1186/s12891-026-09600-8)
Supplement: Supplementary file 1 — Supplementary Material 1. [file 12891_2026_9600_MOESM1_ESM.pdf]

Appendix 1 Professionals

| **Muscle** | **Takeaway** | | **Forward Swing** | | **Acceleration** | | **Early follow-through** | | **Late follow-through** | |
|---|---|---|---|---|---|---|---|---|---|---|
| | **Mean** | **Max.** | **Mean** | **Max.** | **Mean** | **Max.** | **Mean** | **Max.** | **Mean** | **Max.** |
| **Lead Arm** | | | | | | | | | | |
| **ECRB** | | | | | | | | | | |
| Steel | 113.46 | 138.45 | 92.23 | 116.75 | 121.16 | 103.00 | 173.97 | 165.27 | 159.65 | 242.12 |
| Graphite | 107.63 | 134.95 | 88.76 | 117.53 | 117.27 | 109.57 | 174.08 | 160.87 | 167.68 | 244.87 |
| *p-value* | *0.260* | *0.515* | *0.214* | *0.767* | *0.515* | *0.173* | *0.441* | *0.953* | *0.051* | *0.767* |
| **FCU** | | | | | | | | | | |
| Steel | 136.41 | 181.23 | 237.17 | 170.52 | 238.23 | 164.49 | 249.36 | 213.39 | 180.48 | 260.73 |
| Graphite | 131.53 | 178.54 | 230.29 | 171.27 | 233.40 | 168.88 | 241.40 | 206.84 | 167.72 | 252.83 |
| *p-value* | *0.515* | *0.767* | *0.678* | *0.767* | *0.260* | *0.515* | *0.173* | *0.767* | *0.214* | *0.515* |
| **PT** | | | | | | | | | | |
| Steel | 82.96 | 160.42 | 111.36 | 123.98 | 103.97 | 123.08 | 145.48 | 207.46 | 127.20 | 286.27 |
| Graphite | 77.65 | 158.56 | 104.67 | 132.52 | 108.95 | 136.25 | 144.68 | 216.25 | 130.77 | 301.00 |
| *p-value* | *0.086* | *0.594* | *0.374* | *0.441* | *0.953* | *0.767* | *0.859* | *0.859* | *0.767* | *0.441* |
| **BB** | | | | | | | | | | |
| Steel | 37.40 | 87.74 | 66.46 | 71.33 | 118.09 | 81.49 | 172.38 | 174.89 | 168.38 | 286.02 |
| Graphite | 41.45 | 110.81 | 75.97 | 88.04 | 117.70 | 89.04 | 172.84 | 183.59 | 173.17 | 299.03 |
| *p-value* | *0.374* | *0.097* | *0.260* | *0.139* | *0.767* | *0.374* | *0.767* | *0.953* | *0.859* | *0.594* |
| **Trail Arm** | | | | | | | | | | |
| **ECRB** | | | | | | | | | | |
| Steel | 93.91 | 157.1 | 135.81 | 119.91 | 93.39 | 58.07 | 114.17 | 109.77 | 129.11 | 191.73 |
| Graphite | 94.36 | 159.75 | 141.20 | 121.65 | 90.43 | 56.88 | 107.24 | 103.35 | 134.00 | 200.46 |
| *p-value* | *0.314* | *0.441* | ***0.038*** | *0.953* | *0.594* | *0.859* | *0.314* | *0.374* | *0.594* | *0.515* |
| **FCU** | | | | | | | | | | |
| Steel | 87.57 | 118.78 | 89.06 | 93.45 | 150.23 | 146.20 | 224.09 | 248.38 | 164.65 | 285.44 |
| Graphite | 92.20 | 130.29 | 90.28 | 97.68 | 149.97 | 152.48 | 234.28 | 252.21 | 170.67 | 292.00 |
| *p-value* | *0.515* | *0.813* | *0.214* | *0.859* | *0.515* | *0.441* | *0.594* | *0.953* | *0.767* | *0.678* |
| **PT** | | | | | | | | | | |
| Steel | 57.91 | 107.29 | 55.98 | 71.99 | 87.82 | 84.79 | 151.43 | 183.14 | 163.18 | 264.13 |
| Graphite | 58.32 | 118.08 | 52.11 | 71.36 | 86.25 | 85.36 | 158.82 | 184.98 | 160.84 | 262.53 |
| *p-value* | *0.594* | *0.208* | *0.086* | *0.859* | *0.678* | *0.953* | *0.515* | *0.674* | *0.678* | *0.859* |
| **BB** | | | | | | | | | | |
| Steel | 72.13 | 106.41 | 84.51 | 74.26 | 72.34 | 59.22 | 113.68 | 163.71 | 147.58 | 283.92 |
| Graphite | 69.9 | 106.63 | 82.03 | 70.6 | 73.29 | 60.56 | 116.45 | 159.16 | 153.86 | 294.09 |
| *p-value* | *0.260* | *0.767* | *0.173* | *0.374* | *0.953* | *0.594* | *0.953* | *0.953* | *0.173* | *0.441* |

Statistically significant p-values are shown in bold, * indicates a statistically significant decrease in muscle activity with the graphite shaft

Appendix 2 Amateurs

| Muscle | Takeaway | | Forward Swing | | Acceleration | | Early follow-through | | Late follow-through | |
|---|---|---|---|---|---|---|---|---|---|---|
| | **Mean** | **Max.** | **Mean** | **Max.** | **Mean** | **Max.** | **Mean** | **Max.** | **Mean** | **Max.** |
| **Lead Arm** | | | | | | | | | | |
| **ECRB** | | | | | | | | | | |
| Steel | 81.81 | 154.27 | 53.83 | 73.99 | 73.60 | 98.88 | 137.6 | 215.78 | 133.26 | 250.39 |
| Graphite | 82.08 | 151.08 | 55.29 | 77.36 | 74.94 | 94.08 | 144.96 | 215.27 | 131.27 | 259.00 |
| *p-value* | *0.984* | *0.422* | *0.583* | *0.624* | *0.784* | *0.530* | *0.088* | *0.531* | *0.531* | *0.153* |
| **FCU** | | | | | | | | | | |
| Steel | 81.58 | 158.48 | 127.6 | 188.76 | 131.11 | 177.57 | 166.38 | 225.59 | 151.69 | 271.22 |
| Graphite | 80.41 | 155.37 | 125.07 | 185.07 | 129.29 | 176.50 | 165.21 | 214.83 | 145.72 | 268.01 |
| *p-value* | *0.347* | *0.248* | *0.638* | *0.481* | *1.000* | *0.761* | *0.969* | *0.210* | *0.071* | *0.433* |
| **PT** | | | | | | | | | | |
| Steel | 60.27 | 108.77 | 57.38 | 82.85 | 71.97 | 92.04 | 149.56 | 202.34 | 177. 38 | 302.08 |
| Graphite | 58.81 | 101.02 | 54.08 | 77.20 | 73.93 | 91.81 | 145.96 | 189.57 | 162.03 | 288.49 |
| *p-value* | *0.337* | *0.131* | *0.984* | *1.000* | *0.597* | *0.938* | *0.597* | *0.079* | ***0.005*** | ***0.012*** |
| **BB** | | | | | | | | | | |
| Steel | 34.02 | 85.62 | 35.26 | 72.25 | 58.13 | 98.88 | 151.61 | 215.78 | 178.37 | 314.85 |
| Graphite | 35.87 | 87.67 | 36.61 | 72.15 | 56.76 | 94.08 | 148.6 | 215.27 | 176.23 | 312.56 |
| *p-value* | *0.183* | *0.666* | *0.339* | *0.906* | *1.000* | *0.530* | *0.597* | *0.531* | *0.570* | *0.724* |
| **Trail Arm** | | | | | | | | | | |
| **ECRB** | | | | | | | | | | |
| Steel | 86.01 | 186.62 | 79.18 | 129.42 | 58.67 | 83.78 | 99.83 | 145.09 | 119.22 | 224.8 |
| Graphite | 87.64 | 185.20 | 79.61 | 133.07 | 59.45 | 85.03 | 98.18 | 146.44 | 111.42 | 222.57 |
| *p-value* | *0.399* | *0.299* | *0.422* | ***0.027*** | *0.531* | *0.327* | *0.638* | *0.433* | *0.158* | *0.739* |
| **FCU** | | | | | | | | | | |
| Steel | 52.46 | 90.46 | 62.51 | 104.79 | 125.99 | 170.43 | 215.32 | 262.2 | 162.11 | 302.95 |
| Graphite | 53.04 | 91.11 | 61.47 | 102.59 | 121.70 | 164.45 | 215.01 | 265.33 | 161.17 | 306.39 |
| *p-value* | *0.799* | *0.064* | *0.845* | *0.383* | *0.131* | *0.060* | *0.570* | *0.724* | *0.724* | *0.597* |
| **PT** | | | | | | | | | | |
| Steel | 34.87 | 94.55 | 34.47 | 58.47 | 64.72 | 97.03 | 142.66 | 192.76 | 145.27 | 285.54 |
| Graphite | 35.62 | 94.36 | 36.35 | 60.38 | 66.51 | 100.56 | 145.88 | 195.17 | 140.14 | 279.93 |
| *p-value* | *0.583* | *0.791* | *0.445* | *0.394* | *0.481* | *0.232* | *0.367* | *0.531* | *0.203* | *0.456* |
| **BB** | | | | | | | | | | |
| Steel | 35.77 | 105.67 | 49.47 | 83.09 | 65.78 | 94.61 | 147.79 | 199.37 | 163.95 | 309.63 |
| Graphite | 35.67 | 101.37 | 48.44 | 81.51 | 65.44 | 95.74 | 149.82 | 201.53 | 158.55 | 306.73 |
| *p-value* | *0.845* | *0.189* | *0.217* | *0.337* | *0.389* | *0.953* | *0.769* | *0.695* | *0.308* | *0.695* |

Statistically significant p-values are shown in bold, * indicates a statistically significant decrease in muscle activity with the graphite shaft

Appendix 3 Overlap-Grip

| Muscle | Takeaway | | Forward Swing | | Acceleration | | Early follow-through | | Late follow-through | |
|---|---|---|---|---|---|---|---|---|---|---|
| | **Mean** | **Max.** | **Mean** | **Max.** | **Mean** | **Max.** | **Mean** | **Max.** | **Mean** | **Max.** |
| **Lead Arm** | | | | | | | | | | |
| **ECRB** | | | | | | | | | | |
| Steel | 81.84 | 136.79 | 53.51 | 70.02 | 76.18 | 95.60 | 137.54 | 176.27 | 125.32 | 238.10 |
| Graphite | 81.70 | 138.04 | 52.57 | 74.21 | 74.10 | 96.79 | 138.09 | 182.27 | 126.95 | 246.45 |
| *p-value* | *0.927* | *0.670* | *0.212* | *0.274* | *0.394* | *0.648* | *0.738* | *0.362* | *0.484* | *0.330* |
| **FCU** | | | | | | | | | | |
| Steel | 95.52 | 172.92 | 142.47 | 189.61 | 147.13 | 173.85 | 191.23 | 221.1 | 169.58 | 268.37 |
| Graphite | 91.69 | 162.67 | 135.8 | 185.98 | 145.59 | 174.39 | 185.26 | 209.45 | 166.14 | 268.06 |
| *p-value* | *0.094* | *0.055* | *0.094* | *0.346* | *0.503* | *0.648* | *0.073* | *0.191* | *0.465* | *0.693* |
| **PT** | | | | | | | | | | |
| Steel | 65.00 | 122.33 | 58.69 | 77.58 | 58.00 | 82.10 | 126.49 | 186.25 | 154.45 | 285.3 |
| Graphite | 64.92 | 120.31 | 58.07 | 81.68 | 61.93 | 87.66 | 125.64 | 189.22 | 149.14 | 290.63 |
| *p-value* | *0.465* | *0.394* | *1.000* | *0.136* | *0.927* | *0.523* | *0.605* | *0.808* | *0.171* | *0.951* |
| **BB** | | | | | | | | | | |
| Steel | 39.49 | 78.73 | 48.35 | 61.79 | 79.55 | 89.62 | 165.61 | 200.11 | 185.3 | 295.62 |
| Graphite | 40.6 | 88.53 | 50.42 | 65.49 | 78.93 | 85.78 | 163.44 | 203.4 | 182.86 | 305.75 |
| *p-value* | *0.346* | *0.089* | *0.584* | *0.523* | *0.879* | *0.987* | *0.693* | *0.715* | *0.784* | *0.153* |
| **Trail Arm** | | | | | | | | | | |
| **ECRB** | | | | | | | | | | |
| Steel | 91.50 | 191.77 | 78.05 | 118.77 | 55.74 | 65.47 | 92.21 | 126.10 | 110.48 | 205.01 |
| Graphite | 92.65 | 193.39 | 80.44 | 124.71 | 55.90 | 65.59 | 94.44 | 129.16 | 110.68 | 212.52 |
| *p-value* | *0.831* | *0.543* | *0.121* | ***0.023*** | *0.976* | *0.738* | *0.465* | *0.287* | *0.879* | *0.107* |
| **FCU** | | | | | | | | | | |
| Steel | 63.12 | 100.73 | 71.10 | 102.83 | 131.87 | 164.6 | 214.53 | 262.25 | 151.31 | 299.06 |
| Graphite | 65.00 | 105.76 | 67.10 | 98.63 | 125.74 | 156.04 | 210.37 | 262.02 | 147.88 | 302.07 |
| *p-value* | *0.378* | *0.207* | ***0.021*** | *0.181* | *0.083* | *0.101* | *0.248* | *0.761* | *0.484* | *0.927* |
| **PT** | | | | | | | | | | |
| Steel | 37.06 | 86.47 | 32.76 | 57.44 | 62.54 | 94.78 | 136.59 | 192.17 | 137.82 | 277.11 |
| Graphite | 38.40 | 89.23 | 32.36 | 60.63 | 63.24 | 95.65 | 142.19 | 197.08 | 133.05 | 277.78 |
| *p-value* | *0.465* | *0.485* | *0.670* | *0.494* | *0.903* | *0.648* | *0.224* | *0.484* | *0.330* | *0.648* |
| **BB** | | | | | | | | | | |
| Steel | 47.61 | 112.47 | 56.74 | 79.81 | 60.80 | 78.00 | 125.64 | 176.34 | 158.65 | 292.64 |
| Graphite | 46.71 | 108.13 | 54.59 | 78.75 | 61.27 | 81.29 | 129.35 | 177.71 | 154.81 | 299.03 |
| *p-value* | *0.346* | *0.394* | *0.064* | *0.584* | *0.563* | *0.224* | *0.605* | *0.648* | *0.605* | *0.394* |

Statistically significant p-values are shown in bold, * indicates a statistically significant decrease in muscle activity with the graphite shaft

Appendix 4 Interlock-Grip

| Muscle | Takeaway | | Forward Swing | | Acceleration | | Early follow-through | | Late follow-through | |
|---|---|---|---|---|---|---|---|---|---|---|
| | Mean | Max. | Mean | Max. | Mean | Max. | Mean | Max. | Mean | Max. |
| **Lead Arm** | | | | | | | | | | |
| **ECRB** | | | | | | | | | | |
| Steel | 101.18 | 163.96 | 71.09 | 95.00 | 92.75 | 106.84 | 154.79 | 185.69 | 152.94 | 251.15 |
| Graphite | 99.36 | 152.74 | 73.49 | 97.06 | 97.41 | 109.78 | 173.00 | 192.51 | 152.48 | 258.78 |
| *p-value* | *0.778* | ***0.035*** | *0.433* | *0.638* | *0.470* | *0.683* | ***0.016*** | *0.397* | *0.975* | *0.198* |
| **FCU** | | | | | | | | | | |
| Steel | 93.87 | 138.85 | 173.87 | 151.74 | 170.69 | 155.46 | 181.64 | 208.84 | 143.41 | 254.39 |
| Graphite | 94.72 | 137.78 | 169.76 | 146.67 | 162.85 | 151.58 | 180.20 | 201.23 | 128.26 | 241.67 |
| *p-value* | *0.730* | *0.510* | *0.826* | *0.826* | *0.177* | *0.272* | *0.730* | *0.730* | ***0.016*** | *0.245* |
| **PT** | | | | | | | | | | |
| Steel | 64.38 | 119.05 | 89.38 | 115.58 | 108.77 | 125.08 | 170.27 | 226.93 | 174.53 | 312.88 |
| Graphite | 59.37 | 108.44 | 79.58 | 104.28 | 112.43 | 123.73 | 170.20 | 204.14 | 155.25 | 292.71 |
| *p-value* | *0.124* | *0.245* | *0.397* | *0.331* | *0.363* | *0.826* | *0.703* | *0.109* | ***0.022*** | *0.056* |
| **BB** | | | | | | | | | | |
| Steel | 27.82 | 92.07 | 34.89 | 76.68 | 53.15 | 90.68 | 134.96 | 217.92 | 155.97 | 323.15 |
| Graphite | 33.15 | 99.13 | 38.53 | 79.47 | 53.76 | 91.12 | 137.40 | 215.52 | 160.52 | 322.25 |
| *p-value* | *0.084* | *0.363* | *0.594* | *0.826* | *0.638* | *0.875* | ***0.035*** | *0.683* | *0.778* | *0.730* |
| **Trail Arm** | | | | | | | | | | |
| **ECRB** | | | | | | | | | | |
| Steel | 97.99 | 151.29 | 115.08 | 135.30 | 83.28 | 89.35 | 114.64 | 146.20 | 132.54 | 220.08 |
| Graphite | 96.75 | 148.78 | 116.39 | 133.90 | 85.32 | 91.34 | 111.51 | 145.46 | 125.53 | 213.47 |
| *p-value* | *0.551* | *0.198* | *0.433* | *0.594* | *0.300* | *0.470* | *0.730* | *0.826* | *0.331* | *0.300* |
| **FCU** | | | | | | | | | | |
| Steel | 53.51 | 91.56 | 61.82 | 95.52 | 126.02 | 153.79 | 216.58 | 239.69 | 171.79 | 277.75 |
| Graphite | 53.82 | 91.36 | 66.00 | 101.94 | 128.55 | 159.17 | 229.68 | 250.79 | 179.00 | 288.92 |
| *p-value* | *0.433* | *0.975* | *0.221* | *0.245* | *0.730* | *0.925* | *0.300* | *0.397* | *0.683* | *0.397* |
| **PT** | | | | | | | | | | |
| Steel | 39.96 | 86.25 | 41.08 | 56.01 | 71.93 | 80.14 | 141.36 | 173.86 | 154.38 | 257.40 |
| Graphite | 39.68 | 86.25 | 43.51 | 56.59 | 73.96 | 86.07 | 147.20 | 173.68 | 150.16 | 267.91 |
| *p-value* | *0.875* | *0.950* | *0.683* | *0.551* | *0.638* | *0.221* | *0.198* | *0.701* | *0.470* | *0.272* |
| **BB** | | | | | | | | | | |
| Steel | 31.13 | 81.99 | 53.60 | 68.81 | 69.51 | 94.98 | 145.34 | 220.00 | 141.45 | 327.32 |
| Graphite | 30.46 | 81.25 | 53.01 | 63.82 | 68.51 | 90.64 | 150.15 | 216.95 | 138.51 | 313.95 |
| *p-value* | *0.826* | *0.594* | *0.397* | *0.096* | *0.510* | *0.158* | *0.683* | *0.925* | *0.826* | *0.221* |

Statistically significant p-values are shown in bold, * indicates a statistically significant decrease in muscle activity with the graphite shaft

Appendix 5 Baseball-Grip

| Muscle | Takeaway | | Forward Swing | | Acceleration | | Early follow-through | | Late follow-through | |
|---|---|---|---|---|---|---|---|---|---|---|
| | Mean | Max. | Mean | Max. | Mean | Max. | Mean | Max. | Mean | Max. |
| **Lead Arm** | | | | | | | | | | |
| **ECRB** | | | | | | | | | | |
| Steel | 86.13 | 195.58 | 90.91 | 134.73 | 107.10 | 178.03 | 166.91 | 253.77 | 181.49 | 316.23 |
| Graphite | 81.00 | 194.90 | 91.59 | 130.10 | 103.54 | 176.52 | 154.08 | 252.17 | 174.64 | 313.90 |
| *p-value* | *0.109* | *0.593* | *1.000* | *0.109* | *1.000* | *0.593* | *0.593* | *1.000* | *0.593* | *0.593* |
| **FCU** | | | | | | | | | | |
| Steel | 81.86 | 207.58 | 126.43 | 300.28 | 144.92 | 269.97 | 153.53 | 301.55 | 139.53 | 340.17 |
| Graphite | 80.48 | 251.10 | 149.95 | 315.93 | 160.06 | 286.17 | 170.02 | 295.58 | 136.68 | 344.93 |
| *p-value* | *1.000* | *0.109* | *0.109* | *0.109* | *0.109* | *0.285* | *0.109* | *1.000* | *0.593* | *0.593* |
| **PT** | | | | | | | | | | |
| Steel | 73.06 | 111.80 | 60.14 | 93.88 | 103.38 | 197.28 | 217.48 | 226.23 | 215.93 | 332.92 |
| Graphite | 65.92 | 91.20 | 56.28 | 82.52 | 91.38 | 108.05 | 184.86 | 204.25 | 198.79 | 288.72 |
| *p-value* | *0.285* | *0.285* | *0.593* | *0.285* | *0.109* | *1.000* | *0.109* | *0.593* | *1.000* | *0.109* |
| **BB** | | | | | | | | | | |
| Steel | 31.20 | 114.73 | 30.28 | 129.07 | 97.01 | 155.93 | 184.31 | 203.30 | 199.71 | 313.35 |
| Graphite | 29.06 | 97.07 | 39.83 | 136.75 | 83.65 | 156.32 | 159.84 | 210.03 | 189.56 | 302.70 |
| *p-value* | *1.000* | *0.285* | *0.285* | *0.109* | *0.285* | *0.593* | *0.109* | *1.000* | *0.109* | *0.109* |
| **Trail Arm** | | | | | | | | | | |
| **ECRB** | | | | | | | | | | |
| Steel | 93.31 | 223.52 | 90.21 | 155.05 | 70.47 | 121.00 | 132.20 | 179.55 | 153.71 | 299.42 |
| Graphite | 96.29 | 216.08 | 86.36 | 159.02 | 58.85 | 120.17 | 91.85 | 154.23 | 119.02 | 275.75 |
| *p-value* | *0.285* | *0.109* | *1.000* | *0.109* | *0.285* | *1.000* | *0.109* | *0.285* | *0.109* | *0.285* |
| **FCU** | | | | | | | | | | |
| Steel | 71.14 | 91.48 | 79.56 | 129.07 | 153.49 | 220.07 | 241.77 | 325.47 | 207.39 | 397.85 |
| Graphite | 75.14 | 95.23 | 83.55 | 121.23 | 143.58 | 217.67 | 239.98 | 319.17 | 208.09 | 377.90 |
| *p-value* | *0.285* | *0.109* | *0.285* | *0.109* | *0.593* | *0.593* | *1.000* | *1.000* | *1.000* | *0.285* |
| **PT** | | | | | | | | | | |
| Steel | 63.08 | 233.47 | 81.24 | 118.47 | 117.03 | 156.32 | 221.53 | 256.58 | 213.60 | 368.15 |
| Graphite | 63.49 | 242.68 | 80.82 | 109.08 | 115.97 | 160.27 | 206.85 | 250.22 | 209.89 | 349.35 |
| *p-value* | *0.593* | *0.285* | *0.593* | *1.000* | *1.000* | *0.593* | *0.109* | *0.593* | *0.593* | *0.285* |
| **BB** | | | | | | | | | | |
| Steel | 75.71 | 166.27 | 79.60 | 148.38 | 95.88 | 114.08 | 226.68 | 172.68 | 260.48 | 280.23 |
| Graphite | 78.07 | 159.28 | 80.73 | 152.48 | 106.59 | 124.77 | 205.04 | 185.15 | 266.61 | 294.08 |
| *p-value* | *0.593* | *0.593* | *0.593* | *0.593* | *0.109* | *0.593* | *0.109* | *1.000* | *0.285* | *0.593* |

Statistically significant p-values are shown in bold, * indicates a statistically significant decrease in muscle activity with the graphite shaft

Appendix 6 No Preexisting Pain during Golf

| **Muscle** | **Takeaway** | | **Forward Swing** | | **Acceleration** | | **Early follow-through** | | **Late follow-through** | |
|---|---|---|---|---|---|---|---|---|---|---|
| | **Mean** | **Max.** | **Mean** | **Max.** | **Mean** | **Max.** | **Mean** | **Max.** | **Mean** | **Max.** |
| **Lead Arm** | | | | | | | | | | |
| **ECRB** | 91.53 | 150.67 | 66.56 | 86.46 | 89.39 | 103.84 | 145.43 | 174.98 | 139.81 | 239.36 |
| Steel | 90.38 | 148.61 | 66.02 | 87.80 | 89.20 | 105.71 | 150.18 | 179.64 | 137.70 | 248.42 |
| Graphite | *0.682* | *0.633* | *0.439* | *0.927* | *0.733* | *0.649* | *0.255* | *0.305* | *0.452* | *0.194* |
| *p-value* | | | | | | | | | | |
| **FCU** | | | | | | | | | | |
| Steel | 94.17 | 161.99 | 158.36 | 194.15 | 168.89 | 179.93 | 193.92 | 211.88 | 162.09 | 251.62 |
| Graphite | 92.06 | 162.64 | 154.79 | 188.76 | 167.49 | 179.51 | 192.85 | 203.87 | 154.39 | 246.25 |
| *p-value* | *0.399* | *0.554* | *0.554* | *0.412* | *0.733* | *0.785* | *0.964* | *0.399* | *0.080* | *0.509* |
| **PT** | | | | | | | | | | |
| Steel | 71.37 | 116.04 | 80.33 | 100.71 | 91.78 | 102.91 | 160.71 | 186.43 | 173.39 | 277.00 |
| Graphite | 68.15 | 108.00 | 76.07 | 97.24 | 94.20 | 111.48 | 156.79 | 179.93 | 156.67 | 271.29 |
| *p-value* | *0.088* | *0.172* | *0.716* | *0.964* | *0.633* | *0.194* | *0.480* | *0.487* | ***0.002*** | *0.305* |
| **BB** | | | | | | | | | | |
| Steel | 32.75 | 70.21 | 41.36 | 69.91 | 71.91 | 95.50 | 152.37 | 190.34 | 176.78 | 299.31 |
| Graphite | 37.69 | 80.81 | 46.03 | 74.54 | 70.38 | 96.02 | 149.92 | 191.23 | 174.39 | 303.23 |
| *p-value* | ***0.032*** | *0.094* | *0.122* | *0.419* | *0.802* | *0.785* | *0.649* | *0.750* | *0.265* | *0.855* |
| **Trail Arm** | | | | | | | | | | |
| **ECRB** | | | | | | | | | | |
| Steel | 97.31 | 165.78 | 98.60 | 123.57 | 72.52 | 79.33 | 105.91 | 125.41 | 122.42 | 205.54 |
| Graphite | 96.76 | 163.82 | 100.39 | 126.33 | 71.96 | 82.77 | 102.18 | 128.50 | 116.71 | 209.53 |
| *p-value* | *0.649* | *0.356* | *0.145* | *0.187* | *1.000* | *0.145* | *0.927* | *0.750* | *0.339* | *0.600* |
| **FCU** | | | | | | | | | | |
| Steel | 53.71 | 89.24 | 61.47 | 94.72 | 126.03 | 150.21 | 210.71 | 234.00 | 164.06 | 279.05 |
| Graphite | 55.11 | 90.68 | 61.74 | 94.12 | 122.88 | 150.40 | 214.83 | 239.27 | 163.53 | 284.54 |
| *p-value* | *0.964* | *0.855* | *0.569* | *0.368* | *0.116* | *0.909* | *0.820* | *0.600* | *0.495* | *0.820* |
| **PT** | | | | | | | | | | |
| Steel | 40.59 | 102.85 | 40.02 | 60.46 | 75.61 | 86.26 | 153.16 | 173.97 | 162.65 | 270.24 |
| Graphite | 41.77 | 104.85 | 38.70 | 59.18 | 76.11 | 88.22 | 159.66 | 179.22 | 154.37 | 267.19 |
| *p-value* | *0.425* | *0.319* | *0.246* | *0.991* | *0.785* | *0.495* | *0.179* | *0.471* | ***0.032*** | *0.802* |
| **BB** | | | | | | | | | | |
| Steel | 45.13 | 103.00 | 59.74 | 81.14 | 68.04 | 81.83 | 138.79 | 179.32 | 169.01 | 303.04 |
| Graphite | 44.37 | 101.95 | 56.88 | 76.48 | 68.00 | 82.71 | 140.10 | 187.01 | 163.90 | 308.11 |
| *p-value* | *0.569* | *0.847* | ***0.011*** | *0.072* | *0.927* | *1.000* | *0.802* | *0.285* | *0.425* | *0.274* |

Statistically significant p-values are shown in bold, * indicates a statistically significant decrease in muscle activity with the graphite shaft

Appendix 7 Preexisting Pain during Golf

| Muscle | Takeaway | | Forward Swing | | Acceleration | | Early follow-through | | Late follow-through | |
|---|---|---|---|---|---|---|---|---|---|---|
| | Mean | Max. | Mean | Max. | Mean | Max. | Mean | Max. | Mean | Max. |
| **Lead Arm** | | | | | | | | | | |
| **ECRB** | | | | | | | | | | |
| Steel | 82.85 | 150.80 | 52.92 | 76.98 | 72.41 | 110.09 | 146.60 | 209.65 | 137.77 | 269.93 |
| Graphite | 81.88 | 144.75 | 55.37 | 83.13 | 73.42 | 111.05 | 154.61 | 217.83 | 143.59 | 273.11 |
| *p-value* | *0.530* | *0.308* | *0.182* | *0.182* | *0.638* | *0.814* | *0.099* | *0.308* | *0.272* | *0.583* |
| **FCU** | | | | | | | | | | |
| Steel | 93.26 | 167.35 | 138.03 | 162.50 | 123.30 | 162.25 | 164.35 | 248.42 | 149.02 | 309.08 |
| Graphite | 91.56 | 155.79 | 134.65 | 166.12 | 118.26 | 163.78 | 157.84 | 234.41 | 142.00 | 307.38 |
| *p-value* | *0.433* | *0.209* | *0.480* | *0.814* | *0.272* | *0.583* | *0.182* | *0.388* | *0.158* | *0.308* |
| **PT** | | | | | | | | | | |
| Steel | 51.38 | 130.55 | 44.32 | 72.00 | 49.75 | 89.99 | 120.49 | 243.30 | 149.05 | 348.75 |
| Graphite | 51.16 | 127.90 | 40.73 | 71.93 | 52.91 | 79.26 | 119.73 | 232.07 | 151.10 | 337.71 |
| *p-value* | *0.583* | *0.433* | *0.754* | *0.272* | *0.875* | *0.084* | *0.937* | *0.117* | *0.814* | ***0.050*** |
| **BB** | | | | | | | | | | |
| Steel | 39.51 | 123.17 | 44.43 | 77.03 | 70.93 | 93.73 | 165.42 | 244.47 | 174.58 | 323.57 |
| Graphite | 35.82 | 121.05 | 44.14 | 78.48 | 70.69 | 85.76 | 163.69 | 247.60 | 178.23 | 330.13 |
| *p-value* | *0.583* | *0.875* | *0.695* | *0.814* | *1.000* | *0.657* | *0.875* | *0.480* | *0.695* | *0.480* |
| **Trail Arm** | | | | | | | | | | |
| **ECRB** | | | | | | | | | | |
| Steel | 85.97 | 213.12 | 76.33 | 135.93 | 52.41 | 74.89 | 96.41 | 165.51 | 119.18 | 244.94 |
| Graphite | 88.75 | 216.00 | 77.32 | 140.24 | 53.49 | 69.18 | 95.64 | 155.97 | 116.02 | 236.40 |
| *p-value* | *0.272* | *0.814* | *0.347* | *0.308* | *0.530* | *0.388* | *0.814* | *0.937* | *0.695* | *1.000* |
| **FCU** | | | | | | | | | | |
| Steel | 75.86 | 114.54 | 84.86 | 119.78 | 144.10 | 199.44 | 232.66 | 317.64 | 159.47 | 345.60 |
| Graphite | 77.59 | 121.50 | 82.45 | 118.67 | 140.14 | 188.25 | 229.88 | 316.29 | 162.72 | 346.58 |
| *p-value* | *0.480* | *0.136* | *0.433* | *0.906* | *0.480* | ***0.034*** | *0.308* | *0.308* | *0.388* | *1.000* |
| **PT** | | | | | | | | | | |
| Steel | 38.72 | 84.75 | 37.65 | 63.97 | 56.63 | 112.97 | 124.72 | 229.39 | 118.14 | 305.18 |
| Graphite | 38.30 | 87.68 | 42.69 | 71.41 | 58.92 | 117.95 | 123.43 | 224.74 | 122.46 | 296.59 |
| *p-value* | *0.814* | *0.814* | *0.239* | *0.308* | *0.209* | *0.347* | *1.000* | *0.875* | *0.347* | *0.433* |
| **BB** | | | | | | | | | | |
| Steel | 41.20 | 112.45 | 51.79 | 81.01 | 62.84 | 97.90 | 143.20 | 219.43 | 139.86 | 305.73 |
| Graphite | 41.05 | 103.96 | 53.94 | 85.07 | 65.34 | 99.78 | 147.46 | 203.65 | 142.53 | 294.02 |
| *p-value* | *0.875* | *0.084* | *0.347* | *0.480* | *0.158* | *0.638* | *0.433* | *0.209* | *0.638* | *0.099* |

Statistically significant p-values are shown in bold, * indicates a statistically significant decrease in muscle activity with the graphite shaft

Appendix 8 Male Golfers

| **Muscle** | **Takeaway** | | **Forward Swing** | | **Acceleration** | | **Early follow-through** | | **Late follow-through** | |
|---|---|---|---|---|---|---|---|---|---|---|
| | **Mean** | **Max.** | **Mean** | **Max.** | **Mean** | **Max.** | **Mean** | **Max.** | **Mean** | **Max.** |
| **Lead Arm** | | | | | | | | | | |
| **ECRB** | | | | | | | | | | |
| Steel | 93.53 | 147.61 | 67.87 | 87.26 | 86.85 | 100.96 | 149.18 | 180.53 | 142.28 | 250.40 |
| Graphite | 92.06 | 145.28 | 68.03 | 91.09 | 88.02 | 104.41 | 158.72 | 186.55 | 144.29 | 259.40 |
| *p-value* | *0.505* | *0.337* | *0.695* | *0.337* | *0.624* | *0.318* | ***0.015*** | *0.158* | *0.544* | *0.104* |
| **FCU** | | | | | | | | | | |
| Steel | 99.75 | 165.25 | 159.68 | 169.68 | 167.60 | 168.98 | 199.92 | 222.28 | 168.06 | 270.29 |
| Graphite | 97.19 | 157.43 | 152.16 | 166.96 | 162.61 | 168.23 | 194.50 | 208.61 | 158.95 | 265.90 |
| *p-value* | *0.196* | *0.057* | *0.075* | *0.829* | *0.112* | *0.784* | *0.189* | *0.108* | ***0.034*** | *0.422* |
| **PT** | | | | | | | | | | |
| Steel | 65.11 | 117.48 | 76.37 | 101.26 | 82.77 | 101.00 | 146.73 | 199.73 | 165.72 | 296.06 |
| Graphite | 62.77 | 114.33 | 70.78 | 98.18 | 84.90 | 102.70 | 143.89 | 192.84 | 154.12 | 292.22 |
| *p-value* | *0.117* | *0.624* | *0.299* | *0.739* | *0.769* | *0.829* | *0.544* | *0.286* | ***0.014*** | *0.318* |
| **BB** | | | | | | | | | | |
| Steel | 37.31 | 87.87 | 44.02 | 78.94 | 63.78 | 97.69 | 153.62 | 214.44 | 186.76 | 315.51 |
| Graphite | 40.58 | 98.27 | 47.25 | 82.74 | 64.18 | 95.34 | 154.65 | 213.70 | 186.48 | 319.51 |
| *p-value* | *0.085* | *0.056* | *0.481* | *0.512* | *0.557* | *0.959* | *0.281* | *0.845* | *0.638* | *0.845* |
| **Trail Arm** | | | | | | | | | | |
| **ECRB** | | | | | | | | | | |
| Steel | 90.47 | 171.77 | 102.32 | 126.12 | 70.98 | 76.96 | 103.06 | 135.21 | 121.08 | 215.65 |
| Graphite | 89.79 | 171.10 | 102.94 | 129.48 | 71.69 | 73.67 | 103.27 | 131.35 | 118.01 | 212.07 |
| *p-value* | *0.638* | *0.754* | *0.544* | *0.170* | *0.739* | *0.422* | *0.814* | *0.891* | *0.493* | *0.754* |
| **FCU** | | | | | | | | | | |
| Steel | 65.40 | 100.25 | 70.22 | 99.46 | 133.79 | 159.45 | 225.47 | 255.33 | 166.85 | 294.77 |
| Graphite | 66.68 | 105.01 | 69.63 | 100.69 | 131.84 | 158.18 | 228.66 | 261.33 | 168.50 | 296.68 |
| *p-value* | *0.845* | *0.439* | *0.337* | *0.945* | *0.248* | *0.624* | *0.953* | *0.544* | *0.891* | *0.969* |
| **PT** | | | | | | | | | | |
| Steel | 38.73 | 88.12 | 38.72 | 59.23 | 66.06 | 90.42 | 143.80 | 192.63 | 154.25 | 286.49 |
| Graphite | 39.57 | 91.81 | 39.68 | 62.86 | 67.34 | 94.50 | 148.28 | 196.44 | 148.02 | 282.71 |
| *p-value* | *0.505* | *0.133* | *0.984* | *0.210* | *0.739* | *0.196* | *0.272* | *0.289* | *0.100* | *0.695* |
| **BB** | | | | | | | | | | |
| Steel | 39.54 | 99.62 | 53.72 | 79.72 | 62.81 | 89.49 | 140.40 | 201.55 | 158.93 | 311.13 |
| Graphite | 39.50 | 95.82 | 51.76 | 76.61 | 62.54 | 88.03 | 142.56 | 197.83 | 152.80 | 307.96 |
| *p-value* | *0.769* | *0.244* | ***0.036*** | *0.142* | *0.938* | *0.518* | *0.681* | *0.739* | *0.210* | *0.681* |

Statistically significant p-values are shown in bold, * indicates a statistically significant decrease in muscle activity with the graphite shaft

Appendix 9 Female Golfers

| Muscle | Takeaway | | Forward Swing | | Acceleration | | Early follow-through | | Late follow-through | |
|---|---|---|---|---|---|---|---|---|---|---|
| | **Mean** | **Max.** | **Mean** | **Max.** | **Mean** | **Max.** | **Mean** | **Max.** | **Mean** | **Max.** |
| **Lead Arm** | | | | | | | | | | |
| **ECRB** | | | | | | | | | | |
| Steel | 73.10 | 161.38 | 43.85 | 71.03 | 75.52 | 122.11 | 134.06 | 202.08 | 128.57 | 242.07 |
| Graphite | 73.25 | 154.93 | 44.87 | 70.25 | 72.22 | 117.31 | 126.67 | 206.76 | 122.83 | 243.51 |
| *p-value* | *0.953* | *0.859* | *0.594* | *0.953* | *0.214* | *0.515* | *0.374* | *0.859* | *0.515* | *0.953* |
| **FCU** | | | | | | | | | | |
| Steel | 73.81 | 157.92 | 126.68 | 236.24 | 112.53 | 194.06 | 133.83 | 224.79 | 124.08 | 263.94 |
| Graphite | 73.72 | 171.45 | 136.98 | 233.65 | 118.66 | 197.39 | 140.50 | 228.28 | 122.15 | 260.07 |
| *p-value* | *0.953* | *0.260* | *0.110* | *0.594* | *0.173* | *0.594* | *0.314* | *0.594* | *0.374* | *0.374* |
| **PT** | | | | | | | | | | |
| Steel | 66.26 | 130.44 | 45.96 | 60.54 | 66.79 | 92.25 | 155.23 | 216.43 | 167.38 | 307.01 |
| Graphite | 64.00 | 112.73 | 47.15 | 60.27 | 71.18 | 98.74 | 151.81 | 204.98 | 158.03 | 287.76 |
| *p-value* | *0.594* | ***0.028*** | *0.515* | *0.953* | *0.678* | *0.515* | *0.859* | *0.441* | *0.441* | *0.086* |
| **BB** | | | | | | | | | | |
| Steel | 26.07 | 80.02 | 36.27 | 48.31 | 98.63 | 85.60 | 165.48 | 179.52 | 139.46 | 275.85 |
| Graphite | 25.24 | 74.31 | 39.31 | 51.57 | 92.13 | 84.67 | 151.99 | 189.00 | 137.88 | 283.01 |
| *p-value* | *0.859* | *0.441* | *0.139* | *0.678* | *0.086* | *0.953* | *0.110* | *0.374* | *0.678* | *0.441* |
| **Trail Arm** | | | | | | | | | | |
| **ECRB** | | | | | | | | | | |
| Steel | 105.74 | 208.25 | 56.11 | 131.24 | 51.00 | 81.56 | 103.07 | 143.79 | 122.70 | 223.26 |
| Graphite | 110.09 | 208.32 | 60.82 | 134.04 | 48.27 | 95.99 | 89.71 | 155.31 | 111.31 | 236.62 |
| *p-value* | *0.314* | *0.441* | ***0.028*** | *0.441* | *0.953* | ***0.008*** | *0.767* | *0.374* | *0.441* | *0.139* |
| **FCU** | | | | | | | | | | |
| Steel | 42.98 | 85.03 | 62.52 | 111.81 | 123.38 | 184.03 | 189.14 | 272.04 | 148.32 | 313.61 |
| Graphite | 45.23 | 82.44 | 62.17 | 104.24 | 115.06 | 174.06 | 187.26 | 266.01 | 145.33 | 325.44 |
| *p-value* | *0.086* | *0.214* | *0.953* | *0.109* | *0.139* | *0.066* | *0.678* | *0.515* | *0.859* | *0.678* |
| **PT** | | | | | | | | | | |
| Steel | 44.52 | 129.44 | 41.36 | 69.39 | 83.18 | 107.54 | 147.50 | 183.57 | 132.26 | 260.85 |
| Graphite | 44.73 | 126.86 | 40.64 | 62.80 | 83.37 | 106.23 | 150.58 | 180.61 | 133.73 | 252.94 |
| *p-value* | *0.953* | *0.441* | *0.678* | *0.343* | *0.953* | *0.678* | *0.859* | *0.374* | *0.767* | *0.441* |
| **BB** | | | | | | | | | | |
| Steel | 59.13 | 127.26 | 69.89 | 85.84 | 79.11 | 76.87 | 139.14 | 156.19 | 164.90 | 278.76 |
| Graphite | 56.72 | 125.74 | 70.61 | 87.48 | 83.27 | 87.12 | 141.44 | 171.92 | 173.64 | 289.84 |
| *p-value* | *0.173* | *0.767* | *0.953* | *0.953* | *0.314* | *0.066* | *0.953* | *0.260* | *0.139* | *0.314* |

Statistically significant p-values are shown in bold, * indicates a statistically significant decrease in muscle activity with the graphite shaft
